# Operational performance of a programmatic mass drug administration campaign for malaria in southern Mozambique: a comprehensive mixed-methods evaluation of implementation outcomes

**DOI:** 10.1186/s12889-026-27035-7

**Published:** 2026-04-07

**Authors:** Laura Fuente-Soro, Maria Tusell, Jacopo Vecchio, Amancio Nhangave, Khalid Bapu, Christina Riley, Felisbela Materrula, Mércia Dimene, Samira Sibindy, Baltazar Candrinho, Pedro Aide, Caterina Guinovart

**Affiliations:** 1 Barcelona Institute for Global Health, Hospital Clínic - Universitat de Barcelona, Barcelona, Spain; 2 Gaza Provincial Directorate of Health, Xai-Xai, Mozambique; 3 Manhiça Health Research Centre, Manhiça, Mozambique; 4 Akros, Lusaka, Zambia; 5 National Malaria Control Program (NMCP), Ministry of Health, Maputo, Mozambique; 6 National Institute of Health, Maputo, Mozambique; 7 Facultat de Medicina i Ciències de la Salut, Universitat de Barcelona (UB), Barcelona, Spain

**Keywords:** Implementation outcomes, Malaria, Programmatic implementation, Mixed-methods evaluation, Mozambique, Mass drug administration

## Abstract

**Background:**

The World Health Organization (WHO) recommends Mass Drug Administration (MDA) to reduce malaria transmission in low-transmission settings, with a target coverage of at least 80%. To maximize impact, the development of effective and sustainable programmatic implementation strategies is essential. Between December 2022 and February 2023, two rounds of programmatic MDA (pMDA) were implemented in Chidenguele, in Gaza province, Mozambique. The target population was approximately 59,271 individuals. Door-to-door drug distribution, using dihydroartemisinin-piperaquine, was guided by satellite imagery-based mapping.

**Methods:**

A mixed-methods evaluation was conducted to assess operational performance —including acceptability, appropriateness, coverage, feasibility, fidelity, and adoption— using a community household survey (*n* = 770), a health staff survey (*n* = 28), and field observations (*n* = 149).

**Results:**

Among household survey participants eligible for medication during round 2, 96,2% (607/631) *accepted* to participate in the pMDA. All health staff survey participants and 91,3% (703/770) of household survey participants considered taking antimalarials while asymptomatic to be an acceptable preventive measure. All health staff survey participants (28/28) and 84,1% (648/770) of household survey participants felt the intervention was *appropriate* for reducing malaria transmission in the community. Programmatic or contact coverage (proportion of survey participants that received pMDA treatment) reached 73,9% (569/770), while operational coverage (proportion of those present at the time of the intervention that received pMDA treatment) was 90,2% (569/631). All health staff survey participants (28/28) considered the pMDA implementation strategy *feasible* to implement. In terms of *fidelity*, direct observations of pMDA household visits showed that pMDA distribution teams correctly explained the purpose of the visit in 74,5% (111/149) of interactions, requested verbal consent in 86,6% (129/149), and emphasized the importance of completing the treatment in 60,4% (90/149). Health staff survey participants showed strong willingness to *adopt* the procedures, with 86.0% (24/28) and 96.0% (27/28) expressing high commitment in rounds 1 and 2, respectively.

**Conclusions:**

Despite the pMDA delivery strategy being implemented with good operational performance and being well accepted by the community and health staff, reaching the 80% coverage target remains challenging. Sustained collaboration and active engagement from communities, partners, and policymakers, and enhancement of some key outcomes, such as the fidelity of the implementation, are critical to improving coverage and ensuring the success of pMDA campaigns.

**Supplementary Information:**

The online version contains supplementary material available at 10.1186/s12889-026-27035-7.

## Background

In 2022, the World Health Organization (WHO) recommended the implementation of malaria Mass Drug Administration (MDA) to reduce transmission of *Plasmodium falciparum* in very low to low transmission settings, to be conducted in combination with other malaria control strategies such as vector control [1]. MDA for malaria consists of administering a full therapeutic course of antimalarial drugs to the whole population in a defined geographic area at the same time, irrespective of malaria infection status. To ensure that MDA is effective, the WHO recommends that at least 80% of the target population is reached and treated, and that high levels of drug adherence are ensured.

This strategy has already been implemented in several countries worldwide, both in research settings, such as in Mozambique [2], and through programmatic approaches led by National Malaria Control Programmes (NMCP), as in Zambia, the Comoros, and Myanmar [3–6]. Although delivery strategies varied slightly and real-world implementation is more challenging than in research contexts, most malaria MDAs conducted door-to-door in sub-Saharan Africa have achieved treatment coverage rates (individuals treated/target population) falling short of the targets set by the WHO, such as 57% in Zambia and 72.3% in Mozambique [2, 4].

As stated by the WHO, “Implementing mass drug administration is a complex operation requiring a significant investment of resources and careful planning” [7]. Key factors for successful implementation and improved treatment coverage include directly observed therapy (DOT), door-to-door distribution, and enhanced community engagement [7, 8]. Furthermore, robust monitoring should not only evaluate the impact of the intervention on malaria transmission but also assess all contextual factors that may hinder achieving optimal coverage. Because of this, the implementation outcomes as described by Proctor et al. are particularly valuable, as they provide tangible metrics and insights into the real-world implementation of MDA [9]. These outcomes should be measured at all levels (community, health staff and implementation leadership, and policymakers) to inform the optimization of the strategy and generate evidence-based policy change.

For the first time in a low malaria transmission area of Mozambique, between December 2022 and February 2023, two rounds of programmatic MDA (pMDA) with dihydroartemisinin-piperaquine (DHAp) were implemented by the Mozambican NMCP, in collaboration with the Manhiça Health Research Center (CISM) and the Barcelona Institute for Global Health (ISGlobal) as technical partners, in Chidenguele, Gaza province. Following WHO recommendations [8], the delivery strategy included door-to-door distribution in all households plus targeted distribution fixed points, DOT of the first dose of the three-day course, training of local fieldworkers, guides and mobilizers, as well as decentralized distribution. DHAp tablets for the second and third days were left with the participants for self-administration. In addition, to ensure the inclusion of all households in the targeted area, a satellite map platform (RevealⓇ) was piloted to assist fieldworkers in locating the households and monitoring the daily performance of the campaign. Based on lessons learned in the first round, the implementation strategy was optimized for the second round in terms of duration, number of field and community engagement teams, logistics, and other operational aspects to increase coverage. The implementation strategy, programmatic data, coverage and impact of the intervention on the incidence of malaria is reported elsewhere (Tusell et al., manuscript in review). A comprehensive evaluation was conducted in parallel with the pMDA to assess the operational performance and the contextual factors influencing the campaign to identify implementation challenges and inform evidence-based national policies and implementation guidelines.

## Methods

### Study area

The pMDA was conducted in the administrative post of Chidenguele, in Manjacaze district, Gaza province, in southern Mozambique. In 2022, it had an estimated population of 59,271, according to the latest official census estimates. The administrative post is served by seven rural health facilities (HF) and nine community health workers (CHWs).

Malaria transmission intensity in the area is low, with an annual incidence of 121 cases per 1000 population in 2021, according to the routine surveillance data reported through the national DHIS2 system. Transmission is perennial, reaching its peak right after the rainy season, typically from November to March, although this pattern has been delayed in recent years.

Routine malaria control measures in the area consisted of universal bednet distribution campaigns every 3–4 years, annual indoor residual spraying, intermittent preventive treatment during pregnancy, and case management using rapid diagnostic tests and first-line treatment with artemether-lumefantrine.

### Study design and procedures

The operational performance of the pMDA implementation was evaluated using a mixed-methods approach during and after the pMDA campaign, including a cross-sectional community household survey, a health staff survey, and non-participatory observations (Annex 1). Coverage and implementation outcomes, including acceptability, appropriateness, adoption, feasibility, fidelity, and sustainability, using the definitions established by Proctor et al. [9], were evaluated from the community, health providers, coordinators, supervisors, and policy makers’ perspectives. Qualitative evaluations, as well as implementation costs and cost-effectiveness estimates, were also conducted and will be reported elsewhere.

To understand the community’s perspective and evaluate coverage, a cross-sectional community household survey was conducted between February and March 2023, three weeks after the second round of the pMDA (Annex 2). Participants were selected through a multistage cluster sampling process. First, operational areas (clusters) with an equal number of households were defined in RevealⓇ using the field-updated map validated during the pMDA, from which 154 operational areas were randomly selected. Subsequently, five households within each operational area and one individual per household were randomly selected for a total of 770 individuals. In households where no one was found or where the randomly selected individual was absent, up to three attempts were made to ensure those houses or individuals were included. After three attempts, the closest household or another individual in the same household was selected, respectively. Selected individuals were invited to participate and written informed consent was obtained.

To explore perspectives from the health providers, supervisors, coordinators, and policymakers, a health staff survey was also conducted between February and March 2023 (Annex 3). Purposive sampling was used to select 28 participants: seven healthcare workers from all HFs in the administrative post, and 21 health system personnel directly involved in the pMDA implementation, including eight supervisors and 13 coordinators at the district, provincial and national levels. Coordinators included roles such as malaria focal points, chief medical officers, health directors, community engagement officers, pharmacovigilance officers, and statistics focal points, as well as key NMCP personnel at the central level. Participants were selected based on their direct involvement in campaign planning, oversight, and/or implementation to ensure informed responses on operational performance and contextual factors.

Participants were interviewed using standardized electronic questionnaires administered by trained fieldworkers who were not involved in the pMDA implementation. For children, responses were provided by the primary caregiver. For pMDA coordinators and supervisors, the questionnaire was anonymous and self-administered. For the community household survey, sociodemographic information, malaria information (knowledge, use of malaria control strategies, and care-seeking behavior), and pMDA implementation outcomes including coverage, acceptability, adherence, appropriateness, and fidelity were recorded (Annex 2). For the health staff survey, data on knowledge, acceptability, appropriateness, adoption, and sustainability, as well as other factors related to the implementation strategy were collected (Annex 3). Some implementation outcomes were explored by adapting part of the constructs/domains of the Consolidated Framework for Implementation Research (CFIR) to fit the pMDA context [10]. In both surveys, questions included closed- and open-ended questions. Closed-ended questions used a combination of formats, including statements with “I agree/I don’t agree” responses and four- and six-point scales measuring willingness and agreement (Annexes 2 and 3).

Additionally, non-participatory direct observations of the pMDA distribution teams during the campaign household visits were conducted by independent study personnel for both rounds, covering 45 households in round 1 (R1) and 104 households in round 2 (R2), to assess the fidelity to the implementation strategy in the field, including both fieldworker actions and community reactions. A minimum of five observations per day were conducted, with data collection continuing until thematic saturation was reached. All procedures related to the implementation of the campaign -such as the explanation to participants, eligibility screening, and drug administration- were captured using a paper-based checklist that included also open-ended question for further details (Annex 4).

### Data management and statistical analysis

All questionnaires used during the evaluation phase were specifically designed for the purpose of the study. The community household survey questionnaire (Annex 2) was programmed in ODK Collect, while the health staff survey questionnaire (Annex 3) was programmed directly in Research Electronic Data Capture (REDCap) [11]. In both surveys, data were uploaded to a database in REDCap hosted on the CISM server at the end of each day. Data from the paper-based checklists filled out during the non-participatory observations were tabulated into a matrix format using MS Excel (Annex 4).

A sample of 770 participants was calculated for the community household survey to allow us to estimate, using conservative estimates as recommended by the WHO [12], among a population of 60,000 individuals, the different indicators at a prevalence of 50%, with a precision of 5%, a confidence level of 95% and a design effect of 2 to adjust for cluster sampling.

Categorical variables were analyzed as proportions along with their corresponding 95% confidence interval (CI). Statistical analyses were performed using Stata 16.1 (StataCorp. 2020. Stata Statistical Software: Release 16.1. College Station, TX: StataCorp LLC.). Qualitative data from open-ended questions embedded in both survey questionnaires were extracted and coded into a different matrix format using MS Excel. Conceptually similar barriers or improvements were grouped into categories, and any discrepancies were resolved through discussions among the research team, occasionally leading to the reclassification of codes.

### Study outcomes

Implementation outcomes, including acceptability, appropriateness, adoption, feasibility, fidelity, and sustainability, were measured using the definitions established by Proctor et al. [9]. Additional indicators defined in Supplementary Table 1 were also used to evaluate the operational performance of the programmatic implementation. Household- and individual-level coverage, along with adherence to the antimalarials in R2, were estimated. Adherence was self-reported for all ages, but directly observed only for participants older than 14 years given their dosing schedule consisted of a full blister of tablets, which was easier to evaluate accurately. Briefly, acceptability was assessed through questions on participants’ and health staff’s satisfaction and willingness to participate; appropriateness through perceived relevance of the pMDA to community needs; adoption and sustainability through questions on health staff’s intentions and opinions regarding pMDA implementation; feasibility through questions to health staff about the likelihood of successful implementation; and fidelity through direct observation of campaign procedures and reports from community participants (see Supplementary Table 1 for a summary of indicators and data sources).

## Results

### Baseline characteristics of the evaluation participants

The general characteristics of the respondents included in both the community household survey and the health staff survey are presented in Table 1. Among the 770 participants in the community household survey, the majority were female (465/770, 60,4%, CI 56,8% – 63,8%), aged 18 to 59 years (356/770, 46,2%, CI 42,7%–49,8%), and residing in Chidenguele Sede (270/770, 35,1%, CI 31,7%—38,5%).

**Table 1:** Characteristics of the participants included in the community household survey (*n* = 770) and health staff survey (*n* = 28)

	**SURVEY RESPONDENTS**	
	**n**	**%**
Community household survey (*n* = 770)		
Locality		
Chidenguele Sede	270	35,1
Betula	165	21,4
Chicuangue	105	13,6
Dengoine	175	22,7
Mbanze	55	7,1
Age		
< 5 years	68	8,8
5–17 years	205	26,6
18–59 years	356	46,2
> 60 years	133	17,3
Unknown	8	1,0
Gender		
Female	465	60,4
Male	305	39,6
Health staff survey (*n* = 28)		
Age		
Median age (IQR)	34,5 (30,5—39,0)	
Gender		
Female	14	50,0
Male	14	50,0
Role in the MDA campaign		
Healthcare worker in a health facility	7	25
Coordinator/supervisor	21	75
Level of influence		
National	2	7,1
Province	4	14,3
District	22	78,6

In the health staff survey, half of the respondents were female and half male (14/28, 50,0%, 30,6%—69,3%). Most of them were coordinators or supervisors (21/28, 75%, CI 55,1%—89,3%) in the pMDA and were working at the district level (22/28, 78,6%, CI 59,0%—91,7%).

### Acceptability, appropriateness, and adoption of the pMDA strategy

Among participants in the community household survey who reported having been visited by pMDA distribution teams (469 in R1 and 631 in R2), 96% accepted to participate in the pMDA. Specifically, 96,4% in R1 (452/469, CI 94,3%—97,9%) and 96,2% (607/631, CI 94,4%—97,5%) in R2. Among all participants, 91,3% (703/770, CI 89,1%—93,2%) said that it was acceptable to take the medication to prevent malaria even if they were not sick. Out of the 631 participants who reported having been visited, 24 (3,8%, CI 2,5%—5,6%) declined to be treated, and being afraid of the potential side effects was the main reason for refusal (Table 2a). All health staff survey participants (28/28) agreed that it was acceptable to take malaria medication even without being ill to prevent malaria (Table 2b).

**Table 2:** Acceptability and appropriateness of the pMDA campaign among a) community members (from community household survey, *n* = 770), and b) health staff (from health staff survey, *n* = 28)

**a) Community household survey**							
		**Numerator (n)**	**Denominator (N)**	**Rounds**	**n/N**	**Coverage**	**95% CI**
Acceptability	Acceptance rate	Number of surveyed individuals who reported having accepted to participate in the pMDA	Total number of surveyed individuals who reported having been visited by the pMDA teams	Round 1	452/469	96,4%	94,3%—97,9%
				Round 2	607/631	96,2%	94,4%—97,5%
	Refusal rate	Number of surveyed individuals who reported that they refused to take the MDA medication	Total number of surveyed individuals who reported having been visited by the pMDA teams	Round 1	17/469	3,6%	2,1%—5,7%
				Round 2	24/631	3,8%	2,5%—5,6%
	Preventive treatment acceptability	Number of surveyed individuals who think that DHAp is acceptable for prevention, even in a healthy population	Total number of individuals surveyed	Overall	703/770	91,3%	89,1%—93,2%
Appropriateness	Appropriateness	Number of surveyed individuals who think that the MDA campaign could decrease malaria transmission	Total number of individuals surveyed	Overall	648/770	84,1%	81,4%—86,7%

**b) Health staff survey**							
					n/N	%	95% CI
Acceptability	Do you think it’s acceptable to have to take malaria medication even if you’re not ill, to prevent malaria?			Yes	28/28	100%	87,6%—100%^*^
	Do you agree with this statement? If a person does not have malaria, there is no need to give a malaria drug to prevent the disease			Yes	4/28	14,3%	4,0%—32,6%
				No	24/28	85,7%	67,3%—96,0%
Appropriateness	Do you think that an MDA campaign can help reduce malaria in the community?			Yes	28/28	100%	87,6%—100%^*^

^*^One-sided, 97,5% confidence interval

When participants were asked about the perceived fit, relevance or compatibility (appropriateness) of the pMDA as a strategy to decrease malaria transmission in the community, all of the health staff survey (28/28) and the majority (648/770, 84,1%, CI 81,4%—86,7%) of the community household survey respondents agreed that the intervention was appropriate (Table 2a and b).

**Fig. 1:**
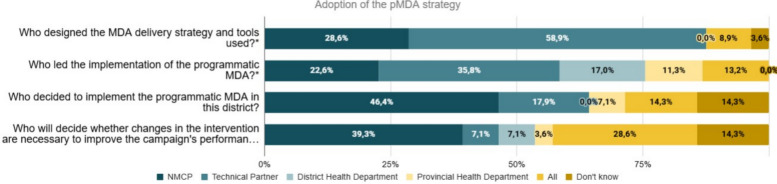
Adoption among health staff (*n* = 28). Questions exploring the adoption of the pMDA implementation strategy were derived from the innovation/intervention characteristics domain of the CFIR framework. * In both questions, the denominator corresponds to the total number of responses that were selected by the health staff in questions where multiple responses could be selected

Regarding the initial intention of health staff to implement the pMDA (adoption), 86% (24/28, CI 67,3—96,0) of the individuals were very well disposed to adopt, collaborate, and comply with the campaign procedures in R1, while this proportion increased to 96% (27/28, CI 81,7–99,9) in R2. Moreover, to better contextualize adoption, the ownership of the intervention and the leadership of the implementation strategy were explored as shown in Fig. 1. Forty-six-point six percent (46,6%, 13/28) referred that the NMCP decided to implement the pMDA in Manjacaze district, and 39,3% (11/28) said that the NMCP would be the one to decide whether changes in the intervention were necessary to improve the campaign’s performance. Further, when asked about who designed the pMDA implementation strategy and the tools used (where multiple responses could be selected), as well as who led the implementation, the most frequent responses indicated the technical partners. Specifically, 35,8% of responses (19/53 of responses selected) identified implementing partners as the designers, and 58,9% (33/56 of responses selected) as the leaders of the implementation (Fig. 1).

### Coverage and feasibility of the pMDA campaign implementation

Results from the community household survey showed that availability coverage at the household level (defined as the proportion of households reached by the pMDA distribution teams) was 64,5% (497/770, CI 61,1%—67,9%) in R1 and 89,1% (686/770, CI 86,7%—91,2%) in R2. At the individual level, the accessibility coverage (individuals visited/individuals surveyed) was 60,9% (469/770, CI 57,4%—64,4%) in R1 and 81,9% (631/770, CI 79,0%—84,6%) in R2. Programmatic or contact coverage (individuals treated/individuals surveyed) in R1 was 54,2% (417/770, CI 50,6%—57,7%) compared to 73,9% in R2 (569/770, CI 70,6%—77,0%). Among those participants who were present during the pMDA visit, the proportion of individuals treated (operational coverage) was 88,9% (417/469, CI 85,7%—91,6%) in R1 and 90,2% (569/631, CI 87,6%—92,4%) in R2. Among those who reported being treated in R2, adherence to the three-day course was reported by 96,1% of individuals (547/569, CI 94,2%—97,6%). Among individuals older than 14 years of age who showed the blister pack, adherence was observed (empty blister) in 94,3% (100/106, CI 88,1%—97,9%) (Table 3).

**Table 3:** Coverage of the pMDA campaign at the community level (from community household survey, *n* = 770)

		**Numerator (n)**	**Denominator (N)**	**Rounds**	**n/N**	**Coverage**	**95% CI**
Coverage at household level	Availability coverage	Number of surveyed individuals who reported that their household was reached by the pMDA campaign^*^	Total number of individuals surveyed	Round 1 Round 2	497/770 686/770	64,5% 89,1%	61,1%—67,9% 86,7%—91,2%
Coverage at the individual level	Availability coverage	Number of surveyed individuals who reported having been reached by a pMDA team^**^	Total number of individuals surveyed	Round 1 Round 2	499/770 686/770	64,8% 89,1%	61,3%—68,2% 86,7%—91,2%
	Accesibility coverage	Number of surveyed individuals who reported having been visited by a pMDA team	Total number of individuals surveyed	Round 1 Round 2	469/770 631/770	60,9% 81,9%	57,4%—64,4% 79,0%—84,6%
	Programmatic or contact coverage	Number of surveyed individuals who reported having received the first dose of DHAp	Total number of individuals surveyed	Round 1 Round 2	417/770 569/770	54,2% 73,9%	50,6%—57,7% 70,6%—77,0%
	Effective coverage	Number of surveyed individuals who reported having received the first dose of DHAp	Total number of individuals surveyed who report having been reached by the distribution team^**^	Round 1 Round 2	417/499 569/686	83,6% 82,9%	80,0%—86,7% 79,9%—85,7%
	Operational coverage	Number of surveyed individuals who reported having received the first dose of DHAp	Total number of individuals surveyed who reported having been visited by the pMDA team	Round 1 Round 2	417/469 569/631	88,9% 90,2%	85,7%—90,6% 87,6%—92,4%
	Reported adherence to drug regimen	Number of surveyed individuals who reported having taken the full dose of DHAp (3 days)	Total number of individuals surveyed	Round 2^***^	547/770	71,0%	67,7%—74,2%
			Total number of individuals surveyed who reported being treated	Round 2^***^	547/569	96,1%	94,2%—97,6%
	Observed adherence to drug regimen	Number of surveyed individuals > 14 years of age who showed the correct blister pack presentation according to their age	Total number of individuals surveyed > 14 years of age	Round 2^***^	100/106	94,3%	88,1%—97,9%

^*^Households reached include households where the field team found someone, and also households that were empty at the moment of the MDA visit. Teams left a sticker on the door, even in households where there was no one

^**^Individuals reached include those who were temporarily absent during the household visit but who were registered as members of the household

^***^Adherence was only measured in Round 2

**Fig. 2:**
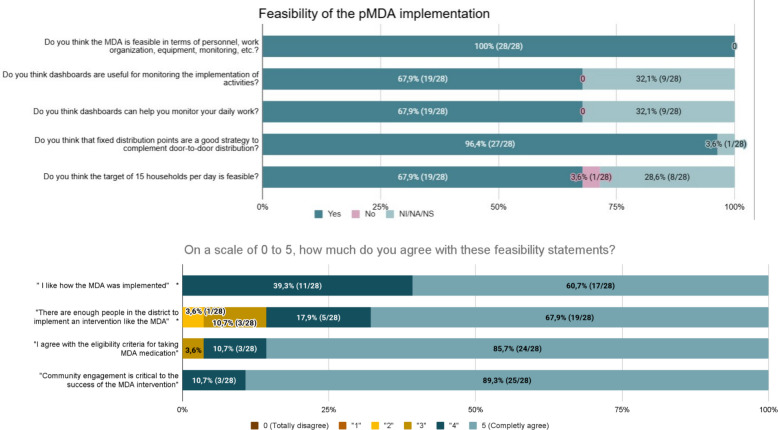
Feasibility of the pMDA implementation among health staff (from health staff survey, *n* = 28). * In both questions, the denominator corresponds to the total number of responses that were selected by the health staff in a question where multiple options could be selected

All health staff who participated in the health staff survey stated that the intervention and the designed implementation strategy, in terms of personnel, work organization, equipment, and monitoring, among other aspects, were feasible to implement (Fig. 2). In addition, on a scale of 0 to 5, 39,3% (11/28, CI 21,5%—59,4%) and 60,7% (17/28, CI 40,6%—78,5%) agreed (“4”) or completely agreed (“5”) with the statement “I like how the MDA was implemented”. When asked about their opinions on whether there were enough personnel in the district to implement an intervention like the pMDA, including drug distribution, supervision, and coordination, whether the eligibility criteria were reasonable, and whether a strong community engagement campaign was needed, the majority of them completely agreed (“5”) with those statements (67,9% (19/28, CI 47,6%—84,1%), 85,7% (24/28, CI 67,3%—96,0%) and 89,3% (25/28, CI 71,8%—97,7%), respectively) (Fig. 2).

### Fidelity of the implementation strategy for pMDA

**Fig. 3:**
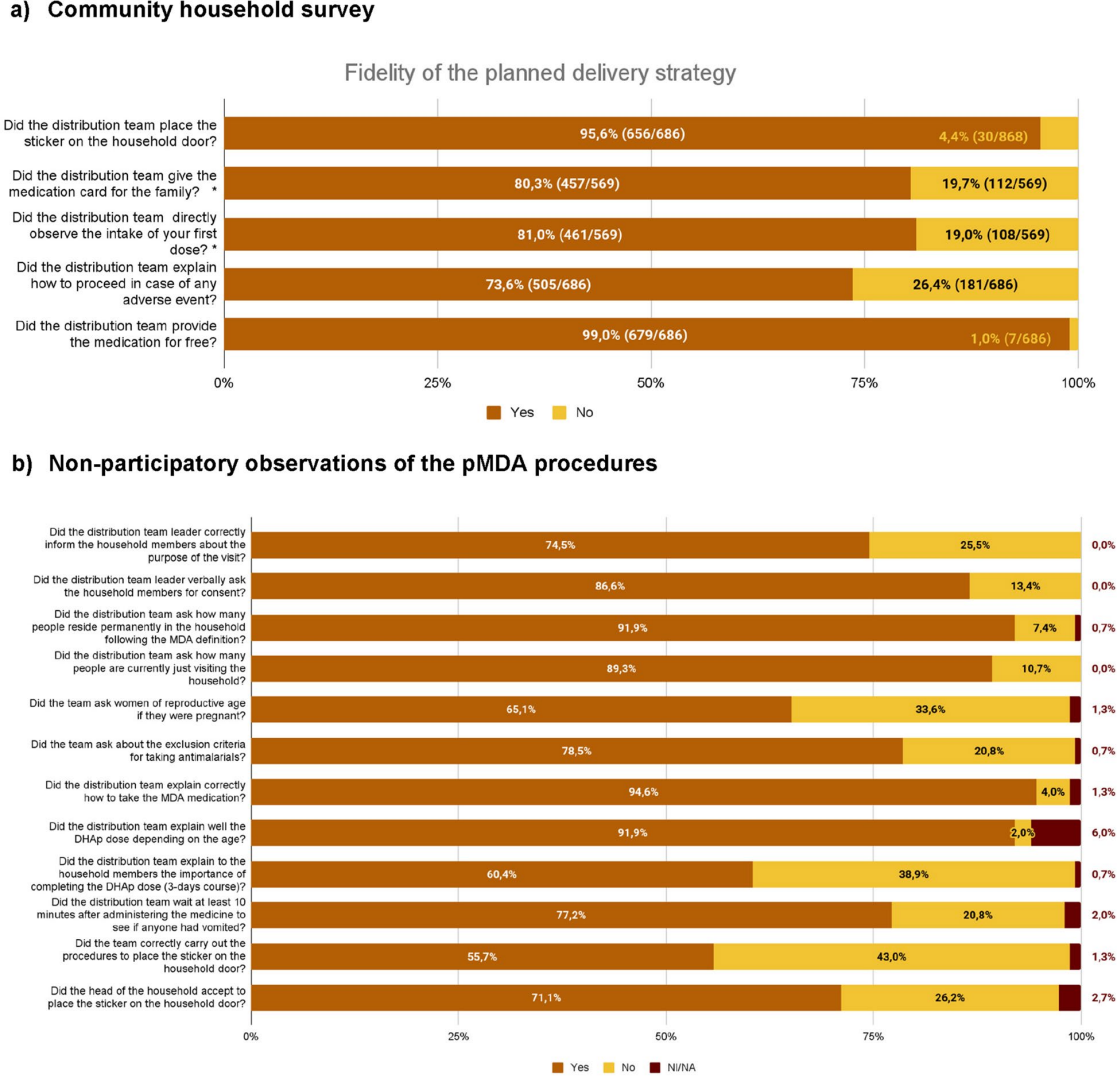
Fidelity of the pMDA campaign to the implementation plan’s procedures was measured at (**a**) community level through the community household survey (*n* = 770), and (**b**) through the non-participatory observations conducted during both rounds of the pMDA (*n* = 154). * This proportion has been calculated among the total number of people treated during the pMDA (*n* = 569)

Of the 686 community survey participants who reported that their household was reached during the second round of the pMDA, 95,6% (656/686, CI 93,8%—97,0%) said that the pMDA distribution team placed a sticker on the door after the visit. Among the 569 who reported being treated, 80,3% (457/569, CI 76,8%—83,5%) said that they received the medication card (a document that was provided to all treated families containing instructions about the dosage for each family member, the date and time of intake for the 2nd and 3rd doses, as well as potential secondary effects and how to act in case of appearance). Nineteen percent (108/569, CI 15,8%—22,4%) of the individuals treated did not take their first dose under the direct supervision of the pMDA distribution team, and in 26,4% (181/686, CI 23,1%—29,8%) of the households the pMDA distribution team did not explain how to proceed in case of an adverse event. Seven individuals out of the total 686 (1%, CI 0,4%—2,1%) reported that someone from the pMDA distribution team asked for money in exchange for the medication, not complying with the procedures described in the implementation plan (Fig. 3a).

The procedures conducted by the pMDA distribution teams during implementation were directly observed in 149 households, 45 in R1 and 104 in R2. The procedures with the lowest fidelity were related to placing a sticker on the doors of households reached by pMDA distribution teams, providing treatment counseling, and verbally screening for pregnancy. Only 55,7% (83/149) of observed visits correctly followed the procedures for placing the household sticker on the door (including asking for consent to place it). In 60,4% (90/149) of observed visits, the pMDA distribution team properly explained to the family the importance of completing the full three-day course of DHAp treatment, and in 65,1% (97/149) of observed visits where there were women of reproductive age, the pMDA distribution team asked if they were pregnant (Fig. 3b).

On the other hand, other procedures were conducted with higher fidelity: the pMDA distribution team correctly explained how to take the medication and the dosage depending on the age of the individual in 94,6% (141/149) and 91,9% (137/149) of the observed visits respectively, verbally asked for consent to conduct the visit and treat them in 86,6% (129/149), explored potential exclusion criteria in 78,5%, (117/149), waited at least 10 min after administering the medication to see if anyone vomited or experienced any side effect in 77,2% (115/149), or informed the family members about the purpose of the visit in 74,5% of the observations (111/149) (Fig. 3b).

### Community engagement, knowledge about the pMDA campaign, and sustainability

Access to information delivered through community engagement activities conducted before the pMDA, as well as knowledge about the campaign, was explored during the community household survey. Among the 770 participants, 88,8% (684/770, CI 86,4%—91,0%) reported having heard about the pMDA, and 24,4% (188/770, CI 21,4%—27,6%) of those reported that they had participated in a community engagement activity. However, when asked whether they knew about the objective of the campaign, only 67,9% (523/770, CI 64,5%—71,2%) reported that they did. More than half of the participants (52,7%, 406/770) stated that the main objective of the campaign was to prevent malaria, while only 9,9% (76/770) specifically mentioned malaria treatment and prevention (Table 4a). The purpose of the intervention was also explored through a multiple-choice question (where multiple responses could be selected) among health staff after completion of both rounds of pMDA. Among the 28 respondents, a total of 68 answers were collected: 29,4% (20/68) of the responses pointed to decreased community malaria transmission, 29,4% (20/68) to malaria elimination, followed by 23,5% referring to malaria prevention (16/68). Almost all of the health staff interviewed (92,9%, 26/28) were very willing to incorporate the pMDA strategy as a routine intervention to prevent malaria in their community, and 64,3% (18/28) of them perceived that the key messages of the pMDA campaign successfully reached the community (Table 4b).

**Table 4:** a) Community engagement campaign outreach and knowledge of the community about the pMDA (from community household survey (*n* = 770)), and b) perceptions among health staff on the community engagement campaign and pMDA sustainability (from health staff survey, *n* = 28)

**a) Community household survey**								
	**Numerator (n)**		**Denominator (N)**	**Rounds**	**n/N**	**Coverage**		**95% CI**
Community engagement coverage	Number of individuals who reported having participated in a community engagement activity		Total number of individuals surveyed	Round 2	188/770	24,4%		21,4%—27,6%
Community MDA knowledge	Number of surveyed individuals who reported having heard about the pMDA		Total number of individuals surveyed	Overall	684/770	89,1%		86,7%—91,2%
	Number of surveyed individuals who were able to mention the pMDA objectives			Overall	523/770	67,9%		64,5%—71,2%
					OBJECTIVES (*n* = 770)^a^: 4,5% (35) malaria treatment 52,7% (406) malaria prevention 9,9% (76) malaria treatment and prevention 3,6% (28) decreasing malaria transmission at the community level			

**b) Health staff survey**								
				n	%		95% CI	
Community engagement coverage	Do you think that the community engagement campaign worked well?	Yes		27	96,4%		81,6%—99,9%	
		No		0	0%		0%—0,1%^b^	
		NI		1	3,6%		0,1%—18,3%	
	Do you think people in your community liked the pMDA campaign?	Yes		27	96,4%		81,6%—99,9%	
		No		0	0%		0%—0,1%^b^	
		NI		1	3,6%		0,1%—18,3%	
	From 0 (strongly disagree) to 5 (strongly agree), how much do you think the key messages about the pMDA campaign successfully reached the entire target population?	5		18	64,3%		44,1%—81,4%	
		4		6	21,4%		8,3%—41%	
		3		4	14,3%		4%—32,7%	
		2		0	0%		0%—0,1%^b^	
		1		0	0%		0%—0,1%^b^	
		0		0	0%		0%—0,1%^b^	
Sustainability	How willing would you be to adopt the pMDA strategy as a routine intervention to prevent malaria?	Very		26	92,9%		76,5%—99,1%	
		Moderately		1	3,6%		0,1%—18,3%	
		Slightly		0	0%		0%—0,1%^b^	
		Not at all		0	0%		0%—0,1%^b^	
		NI		1	3,6%		0,1%—18,3%	

*NI* no information

^a^Multiple choice, more than one option could be selected

^b^One-sided, 97,5% confidence interval

### Suggested improvements & policy translation

When asked about comments or improvements to be incorporated into a future pMDA campaign, 119 out of 770 community respondents provided comments, suggestions, or improvements that could be incorporated into a future pMDA campaign. Forty-six percent of the respondents (55/119) took the opportunity to express their gratitude for the pMDA implementation and for having health campaigns at the community level targeting the entire population. Moreover, 31,1% (37/119) highlighted the long distance to the nearest health facility and the need for a new one in their neighborhood, and 12,6% (15/119) referred that the pMDA should be conducted jointly with other door-to-door health interventions, such as the distribution of bednets or other medicines, such as painkillers.

During the health staff survey, 67,9% (19/28) of the health staff also identified some improvements that, in their opinion, as frontline staff, could increase the pMDA’s operational performance. Almost 37% (7/19) would enhance some logistical aspects, such as reinforcing the number of pMDA distribution teams and the ratio of teams per field supervisor, increasing the fieldworkers’ salaries, or using motorbikes in addition to cars to transport teams to the most distant neighborhoods. Moreover, 26,3% (5/19) suggested validating the satellite maps and the eligible houses before the first round to improve efficiency during the pMDA implementation. Other strategies proposed included improving the community engagement activities, improving the supervision of the key messages delivered in the community, integrating the pMDA with other malaria strategies, such as indoor residual spraying campaigns, or utilizing new therapeutic drugs that do not require a 3-day dose regimen.

Both the programmatic results obtained during the implementation of the pMDA and the findings from this evaluation were presented to the NMCP and other stakeholders during several meetings. A final policy translation workshop was conducted to discuss lessons learned from the pilot implementation and evaluation, refine the implementation strategy in collaboration with the NMCP, and consolidate a final strategy for inclusion in national policy recommendations and implementation guidelines.

## Discussion

This mixed-methods evaluation examined the operational performance of a pMDA implemented in a low-transmission setting in Mozambique. Findings indicate high levels of acceptability, appropriateness, and adoption among health staff. Despite considerable efforts and high household-level availability -particularly in R2 following an optimization of the implementation strategy, including a longer campaign duration and increased number of field and community engagement teams (reported in detail elsewhere; Tusell et al., manuscript in review)- achieving the targeted 80% programmatic or contact coverage remained out of reach, mainly due to individuals who were not reached or visited by pMDA distribution teams, as well as those who refused to participate in the campaign. Under current guidelines, reaching the WHO’s 80% target is unlikely, as some absences and refusals are inevitable. To improve coverage and optimize impact, pMDA distribution teams must reach and visit the majority of targeted individuals, and eligible individuals must be encouraged to participate. A comparison with the programmatic coverage collected during the implementation of the pMDA is reported elsewhere (Tusell et al., manuscript in review).

We identified crucial areas for improvement, highlighting the need for enhanced training on essential procedures to be followed during household visits, to enhance the fidelity of the implementation, and the need to emphasize that the medication treats malaria in case of infection, but also protects for several weeks. Notably, although most campaign procedures were conducted as described in the implementation manual, our findings revealed that for a significant proportion of individuals (19%), the first dose of the treatment was not directly observed by the pMDA distribution teams. Moreover, although pMDA distribution teams were not permitted to leave medication for absent household members, some families requested this. These requests reflected strong community interest in the campaign and gratitude for receiving malaria medication. Some participants expressed a desire to ensure that all family members were treated, including those absent at the moment of the visit, a pattern also observed in previous campaigns in Mozambique [13]. Although DOT has been described as a good practice to increase coverage in MDA campaigns [14, 15], leaving medication for those absent members of the family is not necessarily a bad strategy, and it might actually help reduce the proportion of individuals not being treated due to absence. In addition, this strategy could be reinforced by the high levels of community acceptability, which has been shown previously in trials conducting malaria MDA in other settings, such as Cambodia or Zambia [4, 16], where the majority of the target population expressed their desire to participate in the campaign and take the medication even without being sick. Finally, our results also highlight the importance of having strong community engagement plans and health education campaigns before the MDA implementation to ensure that the objectives of the campaign, or the potential side effects and how to proceed in case of appearance of these, are correctly transmitted to the target population.

Door-to-door campaigns such as MDA are logistically challenging to plan and implement, and require extensive human resources to reach the target population in the minimum number of days. Therefore, robust planning and strong monitoring and evaluation components are essential to ensure high-quality work. Given the limited resources available for thorough personnel training, monitoring pMDA distribution teams on the ground is crucial to track the implementation in real time and address performance deficiencies. In light with this, some participants suggested that conducting a validation round prior to implementation could have ensured that household maps were accurate, highlighting the importance of verifying logistical preparations to improve subsequent rounds. Other improvements such as implementing three rounds, with the first serving as a pilot, could help ensure that most of the target population is reached in at least one of the rounds. This is particularly relevant in rural areas, where limited access to health facilities and long distances make MDA treatment especially critical—a concern expressed by over 30% of community survey respondents.

This study had several limitations. First, potential biases associated with questionnaires, including response and measurement bias should be acknowledged. We tried to address these issues during questionnaire design and implementation and aimed to ensure representative sampling. Additionally, even though the community household survey was conducted by fieldworkers who had not been involved in the implementation of the pMDA, respondents’ perceptions of a link between those teams and the campaign implementers may have also influenced their answers. Similarly, for the health staff survey, although the survey was anonymous, self-administered using an electronic questionnaire, and respondents were encouraged to provide honest feedback, some social desirability bias may have occurred. The use of purposive sampling for the health staff survey may also limit external validity. Moreover, variation in responses among health staff, particularly regarding adoption, may partly reflect differences in participants’ roles and levels within the health system, which could limit their knowledge of higher-level decision-making processes. Finally, some outcomes collected in the community household survey, such as adherence, were assessed only for R2 to minimize recall and memory bias.

The evidence generated from this evaluation was used to inform national policies and refine pMDA implementation guidelines, ultimately enhancing the scalability and success of pMDA campaigns in other eligible regions across Mozambique. By tailoring the implementation strategy to the specific contextual needs of different areas, including population characteristics and geographic distribution of the households, the implementation of pMDA can be significantly improved.

## Conclusions

In conclusion, this comprehensive evaluation of a Mozambican pMDA campaign highlights both the potential and the challenges of implementing and scaling such intervention. While the campaign was generally well-accepted and deemed appropriate by both community members and health facility staff, critical implementation gaps, particularly in achieving the target programmatic or contact coverage, underscore the need for strategic adaptations. Barriers such as absenteeism, and a limited sense of ownership from the NMCP must be addressed to optimize campaign reach and sustainability. Ensuring adequate training, improving community sensitization, and reinforcing the message about the dual role of the intervention, as both preventive and therapeutic, can further strengthen community trust and adherence. Moreover, integrating pMDA with other health interventions and considering additional implementation rounds may enhance coverage, particularly in remote and underserved areas. These insights were essential to inform national policy decisions and refine operational guidelines to successfully implement pMDA in Mozambique and similar settings.

## Supplementary Information

Supplementary Material 1.

## Data Availability

The datasets generated and/or analysed during the current study are available in the Dataverse repository (https://doi.org/10.34810/data2442) upon reasonable request to the corresponding author.

## Abbreviations

CFIR: Consolidated Framework for Implementation Research
CHW: Community Health Workers
CI: Confidence Interval
CISM: Manhiça Health Research Center
DAHp: Dihydroartemisinin-piperaquine
DOT: Directly Observed Therapy
FO: Field Observations
HF: Health Facilities
ISGlobal: Barcelona Institute for Global Health
MDA: Mass Drug Administration
NMCP: National Malaria Control Programmes
pMDA: Programmatic Mass Drug Administration
REDCap: Research Electronic Data Capture
WHO: World Health Organization
