# Supplementary material for: Operational performance of a programmatic mass drug administration campaign for malaria in southern Mozambique: a comprehensive mixed-methods evaluation of implementation outcomes
Source: BMC Public Health. 2026 Apr 7;26:1602. doi: 10.1186/s12889-026-27035-7 (PMC13192075; doi:10.1186/s12889-026-27035-7)
Supplement: Supplementary file 1 — Supplementary Material 1. [file 12889_2026_27035_MOESM1_ESM.zip › ADDITIONAL INFORMATION_Implementation Research Outc_revision 1_CLEAN.docx]

**ADDITIONAL INFORMATION**

**Annex 1. The ASSESS checklist: A comprehenSive tool to Support rEporting and critical appraiSal of qualitative, quantitative, and mixed methods implementation reSearch outcomes**

**Annex 2. Questionnaire used during the community household survey to collect data after the pMDA translated into English.** Original version in Portuguese available upon request. pMDA: programmatic mass drug administration

**Annex 3. Questionnaire used during the health staff survey to collect data after the pMDA translated into English.** Original version in Portuguese available upon request. pMDA: programmatic mass drug administration

**Annex 4. Guideline used during the non-participant observations to collect data during both rounds of the pMDA translated into English.** Original version in Portuguese available upon request. pMDA: programmatic mass drug administration

**SUPPLEMENTARY INFORMATION**

**Table S1.** Summary of the implementation outcomes evaluated: definitions, sources of data, and level of exploration (community, health providers, implementers)

|  |  | **DEFINITION** | | | | **Source of data** |
| --- | --- | --- | --- | --- | --- | --- |
|  |  | **Summary*** | | **Numerator (n)** | **Denominator (N)** |  |
| **ACCEPTABILITY** | **ACCEPTANCE RATE** | Perception among implementation stakeholders that the MDA in Chidenguele was agreeable, palatable, or satisfactory | | Number of surveyed individuals who reported having accepted to participate in the pMDA | Total number of surveyed individuals who reported having been visited by the pMDA teams | - Community Household Survey  - Health staff survey |
|  | **PARTICIPATION REFUSAL RATE** |  |  | Number of surveyed individuals who reported that they refused to take the MDA medication | Total number of surveyed individuals who reported having been visited by the pMDA teams |  |
|  | **TREATMENT REFUSAL RATE** |  |  | Number of surveyed individuals who reported that they refused to take the MDA medication | Total number of surveyed individuals who reported having been visited by the pMDA teams |  |
|  | **PREVENTIVE TREATMENT ACCEPTABILITY** |  |  | Number of surveyed individuals who think that DHAp is acceptable for prevention, even in a healthy population | Total number of individuals surveyed |  |
| **APPROPRIATENESS** | | The perceived fit, relevance, or compatibility of the programmatic MDA for the population of Chidenguele, and/or the perceived fit of the pMDA to reduce malaria transmission | | Number of surveyed individuals who think that the MDA campaign could decrease malaria transmission | Total number of individuals surveyed | - Community Household Survey  - Health staff survey |
| **ADOPTION** | | The intention, initial decision, or action to try or implement the MDA in programmatic mode | | - | - | - Health staff survey |
| **COVERAGE AT HOUSEHOLD LEVEL** | **HH COVERAGE** | The extent to which the pMDA can be successfully implemented | Proportion of households reached by the MDA distribution teams among the number of households surveyed | Number of surveyed individuals who reported that their household was reached by the pMDa campaign** | Total number of individuals surveyed | - Community Household Survey  - Health staff survey |
| **COVERAGE AT INDIVIDUAL LEVEL** | **POPULATION COVERAGE** |  | Proportion of individuals that were reached among survey participants | Number of surveyed individuals who reported having been reached by a pMDA team*** | Total number of individuals surveyed |  |
|  | **VISITATION COVERAGE** |  | Proportion of survey participants who were visited by the distribution team | Number of surveyed individuals who reported having been visited by a pMDA team | Total number of individuals surveyed |  |
|  | **PROGRAMMATIC COVERAGE** |  | Proportion of survey participants who received the treatment | Number of surveyed individuals who reported having received the first dose of DHAp | Total number of individuals surveyed |  |
|  |  |  |  |  |  |  |
|  | **EFFECTIVE COVERAGE** |  | Proportion of survey participants who were reached by the distribution team that received the treatment | Number of surveyed individuals who reported having received the first dose of DHAp | Total number of individuals surveyed who report having been reached by the distribution team*** |  |
|  | **OPERATIONAL COVERAGE** |  | Proportion of survey participants who reported having been visited by the distribution team that received the treatment | Number of surveyed individuals who reported having received the first dose of DHAp | Total number of individuals surveyed who reported having been visited by the pMDA team |  |
|  | **REPORTED Adherence to drug regimen** |  | - | Number of surveyed individuals who reported having taken the full dose of DHAp (3 days) | Total number of individuals treated |  |
|  |  |  |  |  |  |  |
|  | **OBSERVED Adherence to drug regimen** |  | - | Number of surveyed individuals >14 years of age who showed the correct blister pack presentation according to their age | Total number of individuals surveyed > 14 years of age |  |
| **FIDELITY** | | The degree to which the pMDA was implemented as it was prescribed in the original protocol or as the programme developers intended it | | - | - | - Community Household Survey  - No-participatory Direct Observations |
| **SUSTAINABILITY** | | The extent to which a newly implemented intervention, such as the pMD, could be maintained or institutionalized within the NMCP | | - | - | - Health staff survey |
| **COMMUNITY ENGAGEMENT COVERAGE** | | - | | Number of individuals who reported having participated in a community engagement activity | Total number of individuals surveyed | - Community Household Survey  - Health staff survey |
| **COMMUNITY MDA Knowledge** | | - | | Number of surveyed individuals who reported having heard about the pMDA | Total number of individuals surveyed | - Community Household Survey  - Health staff survey |
|  |  |  |  | Number of surveyed individuals who were able to mention the pMDA objectives |  |  |
|  |  |  |  |  |  |  |
| **IMPROVEMENTS** | | - | | | | - Community Household Survey  - Health staff survey |
| * Implementation outcomes definitions have been adapted from Enola et al. Abbreviations: Programmatic Mass Drug Administration (pMDA)  ** Households (HH) reached include those HH where the field team found someone, and also those houses that were empty at the moment of the MDA visit. Teams left a sticker on the door, even in HH, where there was no one.  *** Individuals reached include those who were temporarily absent during the HH visit but who were registered as members of the household | | | | | | |
