# Supplementary material for: Operational performance of a programmatic mass drug administration campaign for malaria in southern Mozambique: a comprehensive mixed-methods evaluation of implementation outcomes
Source: BMC Public Health. 2026 Apr 7;26:1602. doi: 10.1186/s12889-026-27035-7 (PMC13192075; doi:10.1186/s12889-026-27035-7)
Supplement: Supplementary file 1 — Supplementary Material 1. [file 12889_2026_27035_MOESM1_ESM.zip › Annex 2. Community survey_EN.docx]

**Community Household survey to Assess the Programmatic Implementation of MDA**
---------------------------------------------

**PART 1 — Enumerator Information & Household Selection**

Date of visit

__ / __ / ____

Study Number

*ADAM-*|__|__|__|__|

Operational Area Number (Reveal)

____________

Household ID (long Reveal ID)

______________

Name of household head ____________ *(If unknown, write NA.)*

Neighborhood name *[Select from dropdown list]* ______________

Was the house located?

⬜ Yes
⬜ No
⬜ Structure not eligible

*If the house was NOT located or is not eligible, select the nearest neighbouring household (use the Reveal map for identification).
If the house was NOT located or not eligible → end of survey.*

If the house WAS located, record its GPS coordinates:_____________

Was the visit conducted?

⬜ Yes
⬜ No

*If “No”, select the nearest neighboring household (using Reveal map).
If “No”, end of survey.*

*If “Yes”, randomly select 3 household members using paper slips and write their names on the Visit Control List (in the correct order).*

If “No”: Specify the reason

⬜ House destroyed
⬜ Household abandoned or empty after 3 attempts
⬜ Other: _______________________________

**PART 2 — Participant Selection & Socio-Demographic Information**

**Selected Member 1**

Name:
_____________

Was Participant 1 located?

⬜ Yes
⬜ No

If “No”: specify the reason
⬜ Participant absent (select “Absent” only if visited 3 times and not found)
⬜ Participant migrated
⬜ Participant is a visitor, does not permanently reside here
⬜ Deceased
⬜ Other: _______________________

If NOT located: Did the participant take the medication in January/February (Round 2 of MDA)?

⬜ Yes
⬜ No
⬜ Doesn’t know
⬜ Doesn’t want to answer

If NOT located: Did the participant take the medication in November/December (Round 1 of MDA)?

⬜ Yes
⬜ No
⬜ Doesn’t know
⬜ Doesn’t want to answer

*If Participant 1 was NOT located → go to question 24.*

Does the participant (or caregiver) AGREE to participate in the study?

⬜ Yes
⬜ No, refuses

If “No”: Did the participant take the medication in January/February (Round 2)?
⬜ Yes ⬜ No ⬜ Doesn’t know ⬜ Doesn’t want to answer

Did the participant take the medication in November/December (Round 1)?
⬜ Yes ⬜ No ⬜ Doesn’t know ⬜ Doesn’t want to answer

*If the participant 1 refuses → go to question 24.*

*If the participant 1 accepts → sign informed consent and go to question 46.*

**Selected Member 2**

Name:
_____________________

Was Participant 2 located?

⬜ Yes
⬜ No
⬜ Not applicable (not enough persons in household)

*If “Yes”, go to question 31.
If “No”, go to question 27.
If “Not applicable”, end of survey*.

If NOT located: Reason

⬜ Participant absent (after 3 attempts)
⬜ Migrated
⬜ Visitor / not permanent resident
⬜ Deceased
⬜ Other: __________________________

If NOT located: Did the participant take medication in Round 2 (Jan/Feb)?

⬜ Yes ⬜ No ⬜ Doesn’t know ⬜ Doesn’t want to answer

If NOT located: Did the participant take medication in Round 1 (Nov/Dec)?

⬜ Yes ⬜ No ⬜ Doesn’t know ⬜ Doesn’t want to answer

*If Participant 2 NOT located → go to question 35*

Does Participant 2 (or caregiver) AGREE to participate?

⬜ Yes
⬜ No, refuses

*If “No”, ask about taking medication in Round 1 & 2 again (same as above).*

*If Participant 2 refuses → go to question 35.
If accepts → sign consent and go to question 46.*

**Selected Member 3**

Name:
______________________

Was Participant 3 located?

⬜ Yes
⬜ No
⬜ Not applicable (not enough persons in household)

*If Yes, go to question 42.
If No, go to question 38.
If Not applicable → end of survey*.

If NOT located: Reason

⬜ Participant absent (only select “Absent” after 3 attempts)
⬜ Participant migrated
⬜ Visitor / not a permanent resident
⬜ Deceased
⬜ Other: __________________________

If NOT located: Did the participant take the medication in January/February (Round 2)?

⬜ Yes
⬜ No
⬜ Doesn’t know
⬜ Doesn’t want to answer

If NOT located: Did the participant take the medication in November/December (Round 1)?

⬜ Yes
⬜ No
⬜ Doesn’t know
⬜ Doesn’t want to answer

*If Participant 3 NOT located → select the nearest neighbouring household (using Reveal).
End of survey.*

Does Participant 3 (or caregiver) AGREE to participate?

⬜ Yes
⬜ No, refuses

*If “No”: again ask about taking medication in Round 1 & 2 (same questions as above).*

*If the participant refuses → select the nearest neighbouring household (Reveal).
If the participant refuses → end of survey.*

*If the participant accepts → sign informed consent and go to question 46.*

Age

*If the person is older than 1 year, write age in years and check “years”.
If younger than 1 year, write age in months and check “months”.*

Age: _________
⬜ Years
⬜ Months

If age < 18 years: relationship of caregiver to the participant

⬜ Brother/sister
⬜ Father/mother
⬜ Brother-/sister-in-law
⬜ Uncle/aunt
⬜ Cousin
⬜ Nephew/niece
⬜ Not family
⬜ Other: _______________________

Gender

⬜ Female
⬜ Male

Relationship to household head

⬜ Household head
⬜ Spouse
⬜ Son/daughter
⬜ Brother/sister
⬜ Father/mother
⬜ Daughter-in-law
⬜ Brother-/sister-in-law
⬜ Uncle/aunt
⬜ Grandchild
⬜ Stepchild
⬜ Adopted
⬜ Cousin
⬜ Nephew/niece
⬜ Not family
⬜ Other: _______________________

Number of people living in this household *(Those who sleep in the household at least 3 nights per week)* ____________

**PART 3 — HOUSEHOLD & SOCIO-ECONOMIC CHARACTERISTICS**

Type of toilet facility

⬜ Flush toilet connected to septic tank
⬜ Improved latrine
⬜ Traditional improved latrine
⬜ Unimproved latrine
⬜ Shared latrine (with another household)
⬜ No latrine
⬜ Conventional bathroom
⬜ Other: _______________________

Material of main structure walls

⬜ Cement blocks
⬜ Brick blocks
⬜ Wood/metal sheet
⬜ Adobe block
⬜ Reed/Wood/Bamboo/Palm
⬜ Mud sticks
⬜ Tin/Cardboard/Paper/Sack/Bark
⬜ Other: _______________________

Material of main roof

⬜ Cement / slab
⬜ Metal sheet
⬜ Reed
⬜ Thatch
⬜ Other: _______________________

Primary energy source

⬜ Kerosene/paraffin
⬜ Electricity
⬜ Candles
⬜ Solar panels
⬜ None
⬜ Other: ___________________

Cooking fuel

⬜ Firewood
⬜ Charcoal
⬜ Gas
⬜ Electricity
⬜ Kerosene/paraffin
⬜ Other: ___________________

Primary water source

⬜ Bottled water
⬜ Piped water inside house
⬜ Piped water in yard
⬜ Public standpipe
⬜ Protected well/borehole with hand pump
⬜ Unprotected well
⬜ River/lake/pond water
⬜ Rainwater
⬜ Other: ___________________

Do you or your neighbours keep domestic animals on your property?

⬜ Yes
⬜ No

Formal education level of household head

⬜ None
⬜ Primary level 1 (EP1)
⬜ Primary level 2 (EP2)
⬜ Secondary
⬜ Technical / vocational
⬜ Higher education

Formal education level of participant *(If participant <16 years, record education level of primary caregiver.)*

⬜ None
⬜ EP1
⬜ EP2
⬜ Secondary
⬜ Technical
⬜ Higher education

Can the participant (or caregiver, if <16 yrs) read and write?

⬜ Yes
⬜ No

Occupation *(If participant <16 years, record caregiver’s occupation.)*

⬜ Domestic work
⬜ Employed (public/private sector)
⬜ Farmer
⬜ Paid agricultural worker
⬜ Student
⬜ Vendor / trader
⬜ Services
⬜ Other: _____________________
⬜ Not applicable

Religion

⬜ Catholic
⬜ Protestant/Anglican
⬜ Christian (not specified)
⬜ Muslim
⬜ Hindu
⬜ Zionist/Sião
⬜ Animist
⬜ Evangelical / Pentecostal
⬜ Atheist
⬜ Doesn’t know
⬜ Doesn’t want to answer
⬜ Other: ________________________

Did the participant (or caregiver) stop working in the last month due to illness?

⬜ Yes
⬜ No

If “Yes”: number of days stopped working: ______

**PART 4 — MALARIA PREVENTION MEASURES**

Has the house been fumigated in the last 12 months (indoor spraying, PIDOM)?

⬜ Yes
⬜ No

⬜ Don’t know

If NO, why not?

⬜ Team did not come

⬜ No one was home

⬜ They refused

⬜ There has been no spraying campaign in the last 12 months at this location

⬜ Don't know

⬜ Other ______________________

If ‘yes’, look for the date of the last spraying on the label on the door (if the label is not available, enter 01/01/1900). ______________

How many bed nets does the household own? *_______*

How many mosquito nets are hanging in your household? _________

Did you (or your child) sleep under a mosquito net the night before?

⬜ Yes
⬜ No

If “NO”, why not?

⬜ I don't have one

⬜ I don't like them

⬜ It's not hanging up

⬜ It's hot

⬜ You don't need to sleep under a mosquito net

⬜ There are no mosquitoes

⬜ Other:_______________________

Do you or anyone in your household use mosquito coils, repellents, or other products to prevent mosquito bites?

⬜ Yes
⬜ No
Is there tall grass in or near your home during the rainy season?

⬜ Yes
⬜ No

Do you or your child sometimes sleep outdoors?

⬜ Yes
⬜ No

**PART 5 — SEEKING CARE & ACCEPTABILITY**

What disease do you think adults are most at risk of catching in your community? *Do not read the options to the participant and do not influence their answer.*

____________

What do you think adults are most at risk of dying from in your community? *Do not read the options to the participant and do not influence their answer.*

________________

What disease do you think children are most at risk of catching in your community? *Do not read the options to the participant and do not influence their answer.*

____________

What do you think children are most at risk of dying from in your community? *Do not read the options to the participant and do not influence their answer.*

______________

Have you or anyone in your household had a fever in the last 30 days?

⬜ Yes
⬜ No

⬜ Don’t know

⬜ Do not remember

If “yes”, when did you have a fever?

⬜ In the last 24h (today/yesterday)
⬜ More than 2 days ago

⬜ More than one week ago

If “yes”, did the person seek medical attention after the fever? *If the person had a fever in the last 24 hours but answered NO to seeking medical help, refer the person to the nearest health facility.*

⬜ Yes
⬜ No

⬜ Don’t know

If “yes”, where did they seek medical attention?

⬜ Health facility (health centre, health post or hospital)

⬜ APE

⬜ Pharmacy

⬜ Traditional healer

⬜ Other: ________________

If they went to a health centre/primary health care centre/pharmacy, did they get tested for malaria?

⬜ Yes
⬜ No

⬜ Don’t know

If “yes”, was the malaria test positive?

⬜ Yes
⬜ No

⬜ Don’t know

If “yes”, did the person in the household who had malaria take medication from the AMM campaign in January/February (Round 2)?

⬜ Yes
⬜ No

⬜ Don’t know

Do you know what malaria is? *If the person has doubts, use other words to explain it to them.*

⬜ Yes
⬜ No

If “yes”, what do you think is the cause of malaria in this community? *(Multiple choice) (Ask the question to the father/mother/guardian and tick all the boxes that apply to their answer. Do not read, suggest or give any answers to the guardian)*

⬜ Bites from mosquitoes carrying malaria

⬜ Poor diet

⬜ Stagnant water that encourages mosquito breeding

⬜ Filth in homes

⬜ Displeasure of ancestors and spirits

⬜ Heat and sun

⬜ Family conflicts

⬜ Don't know

⬜ Other: ________________________________________

On a scale of 0 to 10, how high do you think the risk is that you and those close to you will contract malaria? *Explain to the participant that 0 is “no risk” and 10 is “very high risk” and that they can choose a value between 0 and 10. If they do not know, write 99.*

*______________*

Compared to 5 years ago, how do you think the problem of malaria has changed in your community?

⬜ Decreased

⬜ No change

⬜ Increased

⬜ Don't know

In the next 5 years, how do you think the number of malaria cases in your community will change?

⬜ Decreased

⬜ No change

⬜ Increased

⬜ Don't know

Over the next five years, how do you think the number of malaria deaths in your community will change?

⬜ Decreased

⬜ No change

⬜ Increased

⬜ Don't know

What do you usually do if you (or your child) have a fever/hot body? *(Multiple choice) (Ask the participant the question and tick all the boxes that apply to their answer. Do not read, suggest or give any answers to the guardian)*

⬜ I go to the nearest health centre to see a health professional

⬜ I go to the local APE (health centre)

⬜ I go to the village healer

⬜ I take a herbal remedy that I make myself (self-medication)

⬜ I take an herbal preparation made by a traditional healer

⬜ I go to the chemist's to buy paracetamol

⬜ I pray to my ancestors and the gods

⬜ I take a bath

⬜ Nothing

⬜ Other: ______________________________________

Do you know how long it takes to walk to the nearest health centre?

⬜ Yes
⬜ No

If “Yes”, how long? __________ ⬜ Hours ⬜ Minutes

When you (or your child) have a fever, how long does it take you to seek medical attention? *(Not including traditional healers)*

⬜ I will go immediately

⬜ 1-3 days

⬜ Between 3 days and 1 week

⬜ More than 1 week

⬜ I don't usually seek medical attention

If “I don't usually seek medical attention”, why not?

⬜ It is far away

⬜ I don't think the health centre/APE will help

⬜ The health centre/APE does not have medicines

⬜ Cost of visiting the health centre

⬜ Have you been to a traditional healer?

⬜ Other: __________________________________________

Do you agree with this statement? ‘Malaria can be serious.’ *(Read the statement to the participant (or caregiver) and ask them if they agree/disagree with it. Read the different options to them)*

⬜ Yes, I agree

⬜ I disagree

⬜ I don't know

Do you agree with this statement? ‘Malaria treatment is important and effective in curing people.’ *(Read the statement to the participant (or carer) and ask them if they agree/disagree with it)*

⬜ Yes, I agree

⬜ I disagree

⬜ I don't know

Do you agree with this statement? ‘Malaria treatment is safe for people.’ *(Read the statement to the participant (or carer) and ask them whether they agree/disagree with it)*

⬜ Yes, I agree

⬜ I disagree

⬜ I don't know

Do you know how malaria can be prevented*? Multiple choice. (Ask the participant or caregiver the question and tick all the boxes that apply to their answer. Do not read, suggest or give any answers to the respondent)*

⬜ Sleeping under mosquito nets

⬜ PIDOM (indoor spraying with insecticides)

⬜ Cleaning the house

⬜ Using anti-malaria medication

⬜ Using repellents and/or insecticides

⬜ Maintaining harmony among community members

⬜ Respecting the law of the ancestors

⬜ Draining stagnant water

⬜ We cannot avoid it if it is our destiny

⬜ Use of medicinal plants

⬜ I do not know

⬜ Other: ________________________________

Do you agree with this statement? ‘I would like to receive (or have my child receive) medication to prevent malaria.’ (*Read the statement to the participant or carer and ask them if they agree/disagree with it.)*

⬜ Yes, I agree

⬜ I disagree

⬜ I don't know

**PART 6 – IMPLEMENTATION OF THE CAMPAIGN**

Have you heard about the mass drug administration campaign for malaria, or “MDA”?

Note: If the participant does not know what MDA is, explain it.

*If “No”, skip to question 110.*

⬜ Yes

⬜ No

If “Yes”, from which sources did you hear about MDA?

⬜ Local leader

⬜ Religious leader

⬜ Health facility worker

⬜ CHW (APE)

⬜ Community mobilizer

⬜ MDA campaign field worker

⬜ Radio

⬜ Printed materials or posters

⬜ Village announcer/mobilizer

⬜ Friends or relatives

⬜ Other: _____________________

Have you ever participated in any sensitization activity related to the mass drug administration campaign for malaria?

*If “No”, skip to question 89.*

⬜ Yes

⬜ No

If “Yes”, which type?

⬜ Health facility talks

⬜ Community meetings

⬜ Mobile mobilization team

⬜ Community activities (theatre, sports events, etc.)

⬜ Church

⬜ Other: _____________________

Do you know the purpose of MDA?

⬜ Yes

⬜ No

If “Yes”, what is it? *Multiple choice. Ask the participant/caregiver and select all that apply. Do not read or suggest answers.*

⬜ To treat malaria

⬜ To prevent malaria

⬜ To treat and prevent malaria

⬜ To reduce malaria in the community

⬜ Other: __________________

Did your household receive a visit from the MDA team during January/February (Round 2)?

*If “No”, help the participant try to recall the visit. If they still answer NO or DON’T KNOW, skip to question 141.*

⬜ Yes

⬜ No

⬜ Don’t know

If “Yes”, is the Round 2 sticker present on the door? *Look for the Round 2 sticker, which should show a date between 17-01-2023 and 02-02-2023.*

⬜ Yes, green

⬜ Yes, yellow

⬜ Yes, red

⬜ No, they never left a sticker

⬜ No, the family removed it

⬜ No, it fell off

If “Yes”, did the distribution team give you a medication card? *Describe the card if the participant does not remember.*

⬜ Yes

⬜ No

⬜ Does not remember

If “Yes”, is the participant showing the card? *Ask the participant to show the card.*

⬜ Yes

⬜ No

Did you take the medication in January/February (during Round 2 of the MDA campaign)?

⬜ Yes

⬜ No

⬜ Don’t know

⬜ Don’t remember

If “No”, why not?

⬜ Not eligible (pregnant, under 6 months of age, or taking a contraindicated medication)

⬜ Refused

⬜ Not at home during the visit

⬜ Other: _____________________

If you answered “refused”, why?

⬜ Did not see the need to take the medication

⬜ Was feeling healthy

⬜ Did not want to take a medication they did not know

⬜ Does not believe the medication prevents malaria

⬜ Treatment duration too long or too complex

⬜ Religious or cultural beliefs

⬜ Interferes with routine habits (e.g., alcohol consumption)

⬜ Fear of the medication or side effects

⬜ Heard negative rumours about the medication

⬜ Does not trust the campaign team

⬜ Does not know the campaign team

⬜ Does not understand what the medication is for

⬜ Was taking other (non-contraindicated) medications and did not want to take many pills

⬜ Other: _____________________

If the answer was “Not eligible”, did the recorder clearly explain why the participant was not eligible for MDA?

⬜ Yes

⬜ No

⬜ Does not remember

If Q114 is “No”/“Don’t know”/“Don’t remember”: Did other household members take the medication?

⬜ Yes

⬜ No

⬜ Don’t know

⬜ Don’t remember

Where did you take the medication during Round 2 (January/February)?

⬜ At my household

⬜ At a fixed distribution point

⬜ Don’t remember

Did the distributor supervise the intake of the first dose?

⬜ Yes

⬜ No

⬜ Don’t remember

Did you understand the information provided by the team about how to take the medication in the days following the visit?

⬜ Yes

⬜ No

If “No”, why not?

⬜ The worker spoke too fast

⬜ The worker spoke in a language I did not understand

⬜ I was not paying attention

⬜ The explanation was unclear

⬜ No explanation was given

⬜ Other: ____________________

If “Yes”, did you take the medication in the following days as explained by the distribution team?

⬜ Yes

⬜ No

⬜ Don’t remember

For how many days did you take the medication?

⬜ Only the first dose in front of the interviewer

⬜ Two (2) days total

⬜ Three (3) days total

⬜ Don’t know

⬜ Don’t remember

⬜ Other: ___________________

If you took only the first dose in front of the interviewer, why?

⬜ Does not believe in the medication

⬜ Not sick

⬜ Felt better after the first dose

⬜ Side effects after the first dose

⬜ Did not like the taste

⬜ Heard rumours against the medication

⬜ Forgot to take it

⬜ Lost the medication

⬜ Saved the medication for when they get sick

⬜ Did not understand the instructions

⬜ Too many pills

⬜ Pills were too large

⬜ Don’t remember

⬜ Other: __________________

Ask the participant to show the box or blister pack from Round 2. Is the blister/box available?

⬜ Yes

⬜ No

If “Yes”, what is the colour of the box or blister?

⬜ Pink

⬜ Orange

⬜ Blue

Number of remaining tablets in the blister:

⬜ 0 ⬜ 1 ⬜ 2 ⬜ 3

⬜ 4 ⬜ 5 ⬜ 6

If “No”, why not?

⬜ Lost

⬜ Left it with someone else

⬜ Discarded

⬜ Other: _____________________

Were you satisfied with how the community distributor interacted with you?

⬜ Yes

⬜ No

⬜ Don’t remember

⬜ Not applicable (not at home during the visit)

Did you understand the campaign team's explanations overall (not only regarding medication intake)?

⬜ Yes

⬜ No

⬜ Don’t remember

⬜ Not applicable (not at home during the visit)

Did the interviewer mention what to do if someone experiences an adverse reaction?

⬜ Yes

⬜ No

⬜ Don’t remember

⬜ Not applicable (not at home during the visit)

Did you experience any adverse reaction during or after taking the medication?

If “No,” “Don’t know,” or “Did not take the medication,” skip to question 140.

⬜ Yes

⬜ No

⬜ Don’t know

⬜ Not applicable, I didn’t take the medication

If “Yes”, what type?

⬜ Skin allergic reaction

⬜ Cardiac issue (palpitations)

⬜ Dizziness

⬜ Vomiting

⬜ Headache

⬜ Joint pain

⬜ General body pain

⬜ Diarrhoea

⬜ Other: ____________________

If “Yes”, when did the adverse reaction start?

⬜ Same day as the first dose

⬜ 1 day after

⬜ 2 days after

⬜ 3 or more days after

⬜ Don’t know

⬜ Don’t remember

If adverse event occurred: Did you inform the distributor or health facility staff?

⬜ Yes

⬜ No

⬜ Don’t remember

If “Yes”: Did you go to the health facility because of the adverse reaction?

⬜ Yes

⬜ No

If “Yes”, how much did you spend in total (transport, medications, consultations, etc.)?

Amount (Meticais): _______________

If “No”, why not?

⬜ Did not know this was an option

⬜ Too far or difficult access

⬜ Did not consider the reaction severe enough

⬜ Afraid of health staff

⬜ Other: _____________________

Did anyone from the distribution team ask you for money in exchange for the medication?

⬜ Yes

⬜ No

⬜ Don’t know

⬜ Not applicable

Did your household receive a visit from the MDA team during Round 1 (December 2022)?

⬜ Yes

⬜ No

⬜ Don’t know

Did you take the medication during Round 1?

⬜ Yes

⬜ No

⬜ Don’t know

⬜ Don’t remember

If “No”, why not?

⬜ Not eligible (pregnancy, <6 months of age, concomitant medication)

⬜ Refused

⬜ Not at home during the visit

⬜ Other: _____________________

If “No”, did other household members take the medication?

⬜ Yes

⬜ No

⬜ Don’t know

⬜ Don’t remember

If “Yes”, are the dates and times recorded on the medication card?

⬜ Yes

⬜ No

⬜ Household does not have a card

If “Yes”, where was the medication taken during Round 1?

⬜ At my household

⬜ At a fixed distribution point

⬜ Don’t remember

If Round 1 visit occurred: Is the Round 1 sticker on the door?

*Sticker should have a date between 01-12-2022 and 13-12-2022.*

⬜ Yes, green

⬜ Yes, yellow

⬜ Yes, red

⬜ No, they never left a sticker

⬜ No, the family removed it

Do you think the MDA campaign can help reduce malaria in the community?

⬜ Yes

⬜ No

⬜ Don’t know

Do you think it is acceptable to take malaria medication even if you are not sick, for prevention?

⬜ Yes

⬜ No

⬜ Prefer not to answer

Has your household received other health campaigns?

⬜ Yes

⬜ No, MDA was the first time a health team visited

⬜ Don’t know

⬜ Prefer not to answer

If “Yes”, which campaigns?

⬜ Polio vaccination

⬜ COVID-19 vaccination

⬜ Mosquito net distribution

⬜ Indoor residual spraying (IRS) for malaria

⬜ Other: __________________

⬜ Don’t know

**CAMPAIGN COVERAGE**

Do you remember how many people were present in the household at the time of the visit in January/February (Round 2)?

⬜ Yes

⬜ No

⬜ Not applicable, did not receive the visit

⬜ Not applicable, not at home

If “Yes”, how many? ________

Among those household members that were present during the visit, was there any pregnant woman, child under 6 months, or person taking contraindicated medication?

⬜ Yes

⬜ No

⬜ Don’t know

⬜ Not applicable, did not receive the visit

⬜ Not applicable, not at home

If “Yes”, how many in total? *If unknown, enter 999*

____________

Do you remember how many of the people present took the medication?

⬜ Yes

⬜ No

⬜ Not applicable, did not receive the visit

⬜ Not applicable, not at home

If “Yes”, how many? _________

Do you remember how many of those who took the medication completed the 3-day regimen?

⬜ Yes

⬜ No

⬜ Not applicable, did not receive the visit

⬜ Not applicable, not at home

If “Yes”, how many? ____

Did anyone in the household take the medication at a fixed distribution point during Round 2?

⬜ Yes

⬜ No

⬜ Don’t know

If “Yes”, how many? _____

Were you satisfied with being reached by the MDA campaign?

⬜ Yes

⬜ No

⬜ Not applicable

⬜ Not at home

Do you have any comments or suggestions about the campaign (e.g., anything that could be improved or that you did not like)? ________________________
