# Supplementary material for: Operational performance of a programmatic mass drug administration campaign for malaria in southern Mozambique: a comprehensive mixed-methods evaluation of implementation outcomes
Source: BMC Public Health. 2026 Apr 7;26:1602. doi: 10.1186/s12889-026-27035-7 (PMC13192075; doi:10.1186/s12889-026-27035-7)
Supplement: Supplementary file 1 — Supplementary Material 1. [file 12889_2026_27035_MOESM1_ESM.zip › Annex 3. Health staff survey_EN.docx]

**Health Workers Survey to Assess the Programmatic Implementation of MDA**
---------------------------------------------

**PART 1 — PARTICIPANT INFORMATION AND DEMOGRAPHICS**

Date of visit:
__ / __ / ____

Study Number:
ADAMHF–___

Was the participant on the list located?
[ ] Yes [ ] No

If “No”, specify the reason:
[ ] Impossible to contact
[ ] Participant not found (after 3 attempts)
[ ] Other: ___________________________

*If “No”, proceed to the next participant. If “No”, end of survey.*

Does the participant AGREE TO PARTICIPATE?
[ ] Yes [ ] No (refusal)

*If “Yes”, sign 2 copies of the informed consent and leave 1 with the participant.
If “No”, invite the next participant. End of survey.*

Age (years): ___

Gender:
[ ] Female
[ ] Male

Type of professional:
[ ] Health professional
[ ] Campaign implementer

Work level:
[ ] Central level
[ ] Provincial level
[ ] District level

If “District level”: Locality: ______________________

If “Health professional”, profession:
[ ] Medical doctor
[ ] Medical/Health technician
[ ] Nurse
[ ] Pharmacist
[ ] Pharmacy technician
[ ] APE (Community Health Worker)
[ ] Administrative staff
[ ] Other: ______________________

If “Campaign implementer”, position:
[ ] District malaria focal point
[ ] Provincial malaria focal point
[ ] District chief medical officer
[ ] Provincial chief medical officer
[ ] District Health Director
[ ] Provincial Health Director
[ ] District community engagement officer
[ ] Provincial community engagement officer
[ ] National PNCM focal person
[ ] Pharmacovigilance officer
[ ] District statistics unit
[ ] Health post/head of locality
[ ] Other: ______________________

Year started in this position: ____

Do you work in a Health Facility?
[ ] Yes [ ] No

If yes, name of the Health Facility: ___________________

In this health facility, is staff turnover generally high or low?
[ ] Staff is stable
[ ] High turnover

---------------------------------------------
**PART 2 — ACCEPTABILITY AND ADOPTION**

In the MDA, were you a coordinator/supervisor?
[ ] Yes, coordinator
[ ] Yes, supervisor
[ ] No

Before implementation, had you heard of MDA?
[ ] Yes
[ ] No

Do you know the purpose of MDA?
[ ] Yes
[ ] No

If yes, what is it? (*Multiple choice*)
[ ] Treat malaria
[ ] Prevent malaria
[ ] Treat and prevent malaria
[ ] Reduce malaria in the community
[ ] Contribute to malaria elimination
[ ] Other: _____________

In your opinion, who designed the strategy/tools for MDA? (*Multiple choice*)
[ ] National Malaria Control Programme
[ ] Manhiça Health Research Center (CISM)
[ ] ISGlobal
[ ] District Health Directorate
[ ] Provincial Health Directorate
[ ] All in consortium
[ ] Don’t know

In your opinion, who led the implementation of MDA? (*Multiple choice*)
[ ] National Malaria Control Programme
[ ] CISM
[ ] ISGlobal
[ ] District Health Directorate
[ ] Provincial Health Directorate
[ ] All in consortium
[ ] Don’t know

Why is MDA being implemented in this district?
[ ] District meets transmission criteria
[ ] Selected by National Malaria Programme
[ ] Malaria is a serious problem in the district
[ ] Other: _____________

Who decided to implement MDA in this district?
[ ] National Malaria Control Programme
[ ] CISM
[ ] ISGlobal
[ ] District Health Directorate
[ ] Provincial Health Directorate
[ ] All in consortium
[ ] Don’t know

How was the decision made to implement MDA?
[ ] Annual partner meeting
[ ] Regular planning meetings
[ ] I received no information
[ ] Based on national programme priorities
[ ] Other: _____________

Do you think MDA can help reduce malaria in the community?
[ ] Yes
[ ] No
[ ] Don’t know

Do you think it is acceptable to take malaria medicine even if you are not sick, for prevention?
[ ] Yes
[ ] No
[ ] Prefer not to answer

Do you agree with this statement:
“If a person does not have malaria, there is no need to give malaria medication to prevent the disease”?
[ ] Yes, I agree
[ ] I do not agree
[ ] Don’t know

What was your reaction when you were told you would participate in MDA implementation?
[ ] Positive
[ ] Negative
[ ] Don’t know

If negative, why?______________________________________________

If involved in MDA — How willing were you at the beginning to follow procedures in Round 1?
[ ] Very willing
[ ] Quite willing
[ ] Slightly willing
[ ] Not willing

If slightly/not willing, why?
[ ] Did not think strategy was useful
[ ] Did not receive enough information
[ ] Other work / unavailable
[ ] Procedures were difficult
[ ] Other: _____________

If involved in MDA — How willing were you to follow procedures in Round 2?
[ ] Very willing
[ ] Quite willing
[ ] Slightly willing
[ ] Not willing

If slightly/not willing, why?
[ ] Did not think strategy was useful
[ ] Did not receive enough information
[ ] Other work / unavailable
[ ] Procedures were difficult
[ ] Other: _____________

If involved in MDA — How willing would you be to adopt MDA as a routine malaria prevention intervention?
[ ] Very willing
[ ] Quite willing
[ ] Slightly willing
[ ] Not willing

If slightly/not willing, why?
[ ] Not useful / ineffective
[ ] Difficult to implement
[ ] Not compatible with other routine activities
[ ] Not a priority compared to other activities
[ ] Other: _____________

Do you think MDA is feasible in terms of staff, organization, equipment, supervision, etc…?
[ ] Yes
[ ] No

If no, what is the main challenge?
[ ] Recruitment of field workers
[ ] Training of field workers
[ ] Work organization
[ ] Logistics (vehicles, etc.)
[ ] Materials and equipment
[ ] Supervision and coordination
[ ] Medicine supply
[ ] Digital/field tools
[ ] Other: _____________

In your opinion, what advantages do you think MDA has over other malaria prevention interventions (IRS, mosquito nets, IPTp, etc.)? _______________________

In your opinion, what disadvantages do you think MDA has in comparison with other malaria prevention interventions (IRS, mosquito nets, IPTp, etc.)? _______________________

During implementation, did you use the dashboards to monitor activities?
[ ] Yes
[ ] No

If yes, did you access the online tool yourself or did someone show you the dashboards/data?
[ ] I accessed it myself
[ ] Someone showed me
[ ] Both

If no, why not?
[ ] I was not aware of the dashboards
[ ] I don’t know how to use them
[ ] I don’t think they are useful
[ ] I had no time to access them
[ ] They were already discussed in review meetings
[ ] Other: ______________________

Do you think dashboards are useful for monitoring activity implementation?
[ ] Yes
[ ] No

Do you agree with the dashboard design?
[ ] Yes
[ ] No

If no, why?__________________________________________________

Did the dashboards meet your expectations?
[ ] Yes
[ ] No

If no, why? __________________________________________________

Do you think the dashboards can help you monitor your daily work?
[ ] Yes
[ ] No

If no, why?__________________________________________________

---------------------------------------------
**PART 4 — IMPLEMENTATION OF THE INTERVENTION**

On a scale from 0 to 5, how much did you like the way MDA was implemented?
*Explain that 0 means “Did not like it at all” and 5 means “Liked it very much”.*
Score: __

If score 0–3, why?__________________________________________________

Would you change or improve anything?
[ ] Yes
[ ] No

If yes, what changes or improvements would help MDA work better? _______________________

Do you think you would be able to make these changes?
[ ] Yes
[ ] No

Why?__________________________________________________

In your opinion, who should decide (or what should the decision process be) if changes are needed to improve campaign performance?__________________________________________________

Are there any components of the intervention that, in your opinion, should NOT be changed?
[ ] Yes, some components should not be changed
[ ] Everything could be changed

If yes, which components should NOT be changed?______________________________________________

On a scale from 0 to 5, how much do you agree with this statement:
“There is enough personnel in the district to implement MDA.” *(0 = strongly disagree, 5 = fully agree, 6 = don't know)*Score: __

If 0–3, why?__________________________________________________

On a scale from 0 to 5, how much do you agree with this statement:
“I agree with the eligibility criteria for MDA/rfMDA medication.” *(0 = strongly disagree, 5 = fully agree, 6 = don't know)*
Score: __

If 0–3, why?__________________________________________________

If 0–3, which criteria do you think should change? (*Multiple choice*)
[ ] Children under 6 months
[ ] Pregnant women
[ ] Anyone with fever or fever history in last 24 hours
[ ] People with known cardiac problems
[ ] Severely ill individuals
[ ] People allergic to artemisinin or piperaquine
[ ] People who took antimalarials in the last 15 days
[ ] People who took contraindicated medications in last 7 days

On a scale from 0 to 5, how much do you agree with this statement: “Community engagement is critical for the success of the intervention.” (*0 = disagree fully, 5 = agree fully, 6 = don't know)*Score: __

If 0–3, why? __________________________________________________

On a scale from 0 to 5, how much do you agree with: “The materials provided for the campaign (bags, T-shirts, etc.) were adequate and sufficient.” (*0 = disagree fully, 5 = agree fully, 6 = don't know)*Score: __

If 0–3, why?__________________________________________________

On a scale from 0 to 5, how much do you agree with: “The registration and monitoring tools used during ROUND 1 (electronic surveys, paper forms, etc.) were adequate and sufficient.”
Score: __

If 0–3, why? __________________________________________________

On a scale from 0 to 5, how much do you agree with:
“The registration and monitoring tools used during ROUND 2 were adequate and sufficient.”
Score: __

If 0–3, why? __________________________________________________

On a scale from 0 to 5, how much do you agree with:
“The mapping tool ‘Reveal’ used for implementation was useful and worked well in the field.”
Score: __

If 0–3, why? __________________________________________________

On a scale from 0 to 5, how much do you agree with:
“The adverse event monitoring system used during the campaign was adequate and sufficient.”
Score: __

If 0–3, why? __________________________________________________

Do you think fixed distribution points are a good strategy to complement door-to-door distribution?
[ ] Yes
[ ] No
[ ] Don’t know
[ ] Not applicable

If no, why? __________________________________________________

If yes, how many days of fixed-point distribution would be ideal?
___ days

When would you place these fixed points?
[ ] At the end of the campaign
[ ] On Sundays
[ ] On holidays
[ ] Other: ____________

Do you think the target of 15 households per day is sufficient, too high, or too low?
[ ] Sufficient
[ ] Too high
[ ] Too low
[ ] Don’t know

Did you receive training in MDA?
[ ] Yes
[ ] No

If yes, how many times were you trained?
__ times

If yes, were you satisfied with the training?
[ ] Yes
[ ] No

If no, why?__________________________________________________

In terms of preparation, do you think you were well prepared and supported to implement the intervention?
[ ] Yes
[ ] No
[ ] Don’t know

If no, what additional support was needed?__________________________________________________

During preparation and implementation, were you satisfied with the role and support of:

National Malaria Control Programme (NMCP)?
[ ] Yes
[ ] No
[ ] Don’t know

If no, what could be improved?__________________________________________________

Manhiça Health Research Center (CISM – technical/logistics partner)?
[ ] Yes
[ ] No
[ ] Don’t know

If no, what could be improved?__________________________________________________

ISGlobal (technical partner)?
[ ] Yes
[ ] No
[ ] Don’t know

If no, what could be improved?__________________________________________________

Provincial Health Directorate?
[ ] Yes
[ ] No
[ ] Don’t know

If no, what could be improved?__________________________________________________

District Health Directorate?
[ ] Yes
[ ] No
[ ] Don’t know

If no, what could be improved?__________________________________________________

Do you think the community mobilisation campaign worked well?
[ ] Yes
[ ] No
[ ] Don’t know

If no, what could be improved?__________________________________________________

On a scale from 0 to 5, how much do you agree with:
“The key messages about the MDA campaign reached the entire population of the Chidenguele Administrative Post.”*(0 = strongly disagree, 5 = fully agree, 6 = don’t know)*
Score: __

If 0–3, why?__________________________________________________

Regarding the community mobilisation campaign, were you satisfied with the role of World Vision (community engagement partner)?
[ ] Yes
[ ] No
[ ] Don’t know

If no, what could be improved?__________________________________________________

What do you suggest to improve the next MDA campaign?____________________________________

Do you think people in your community liked the MDA intervention?
[ ] Yes
[ ] No
[ ] Don’t know

If no, why not?__________________________________________________

What do you suggest as solutions to increase acceptance of the intervention?
__________________________________________________

Do you have any additional questions, suggestions, or comments?_____________________________
