# Supplementary material for: Operational performance of a programmatic mass drug administration campaign for malaria in southern Mozambique: a comprehensive mixed-methods evaluation of implementation outcomes
Source: BMC Public Health. 2026 Apr 7;26:1602. doi: 10.1186/s12889-026-27035-7 (PMC13192075; doi:10.1186/s12889-026-27035-7)
Supplement: Supplementary file 1 — Supplementary Material 1. [file 12889_2026_27035_MOESM1_ESM.zip › Annex 4. Non participant observation guide_EN.docx]

**Non-Participant Observation (NPO)**

---------------------------------------------------

NPO Location: ___ NPO Number: ___ Date: __-__-____ Observer: ___

INSTRUCTIONS

1. This Non-Participating Observation (NPO) is related to the study “Massive and targeted administration of antimalarial drugs to promote malaria control and/or elimination in Mozambique: accelerating programme implementation and policy translation (ADAM-IR)”.
2. This NPO aims to evaluate the procedures of the Mass Drug Administration (MDA) campaign at the Chidenguele administrative post (Manjacaze district) in order to directly capture actions and reactions related to the implementation strategy, including drug administration and all procedures involved in eligibility screening.
3. The NPOs will take place in the homes of participants where the MDA implementation team will be working.
4. Use one form for each location visited. Therefore, at the end of the day, you will have as many forms as visits made.
5. If you detect any situation that warrants clarification or follow-up investigation, write this in the comments section and request authorisation (through Informed Consent) to talk to people about this issue.
6. Informed consent (verbal) with the ‘neighbourhood secretary’ will be requested before activities begin in a given neighbourhood.

--------------------------------------------

1. **NPO INFORMATION**

Date of NPO: __-__-____

NPO Location:

[ ] Household

[ ] Fixed distribution point (specify): ________

Start Time: __:__ End Time: __:__

Team composition:

[ ] 1 Field worker

[ ] 2 Field workers

[ ] Other: ____________

Transport used:

[ ] Walking

[ ] Car

[ ] Motorbike

[ ] Other: ____________

Reference health facility:

[ ] H.F. Chidenguele [ ] H.F. Chizavane [ ] H.F. Incadine

[ ] H.F. Betula [ ] H.F. Ndolene [ ] H.F. Dengoine

[ ] H.F. Mussavelene

--------------------------------------------

1. **OBSERVED PROCEDURES — HOUSEHOLD**

Did the team leader copy the household number from the map to the survey?

[ ] Yes [ ] No

Residents ≥6 months reported: ___

Residents ≥6 months found: ___

Is the household head present?

[ ] Yes [ ] No

If not, reason:

[ ] Away for work

[ ] Away for several days

[ ] Long‑term migration

[ ] Will return later

[ ] Other: ____________

If not , Who responded on behalf of the household head? (Relationship) __________________

Did the team correctly explain the purpose of the visit?

[ ] Yes [ ] No

Did the team verbally request consent?

[ ] Yes [ ] No

Did the household head/delegate allow entry?

[ ] Yes [ ] No

If no, explain: _________________________________________

Did the team ask how many people permanently live in the household (≥4 nights/month)?

[ ] Yes [ ] No

Did the team ask how many visitors are currently present?

[ ] Yes [ ] No

--------------------------------------------

**WOMEN OF REPRODUCTIVE AGE**

Did the team ask if women of reproductive age were pregnant?

[ ] Yes [ ] No

Pregnant women reported: ___

Women 12–49 not reporting pregnancy: ___

--------------------------------------------

**ANTIMALARIAL ADMINISTRATION**

Did the team ask about exclusion criteria?

[ ] Yes [ ] No

Did the team explain correctly how to take the medication?

[ ] Yes [ ] No

Incorrect/confusing messages observed: _________________________________________

Participants who received antimalarial: ___

Participants who took it in presence of team: ___

Participants who refused: ___

Refusals (gender + age):

1. [ ] M [ ] F Age ___

2. [ ] M [ ] F Age ___

3. [ ] M [ ] F Age ___

4. [ ] M [ ] F Age ___

Reasons for refusal: _________________________________________

Did the team explain correct dosages by age?

[ ] Yes [ ] No

Confusing dosage messages: _________________________________________

Did the team explain importance of completing DHAp dose?

[ ] Yes [ ] No

--------------------------------------------

**END OF VISIT**

Did the team complete the daily drug‑distribution control sheet?

[ ] Yes [ ] No

If Round 1: Did they provide the Medication Card?

[ ] Yes [ ] No [ ] Not applicable

Did they wait at least 10 minutes to check for vomiting?

[ ] Yes [ ] No

Did they mark fingers with indelible ink (treated + ineligible)?

[ ] Yes [ ] No [ ] Not applicable

Did they correctly place the household sticker?

[ ] Yes [ ] No [ ] Not applicable

Did the household head accept the sticker?

[ ] Yes [ ] No [ ] Not applicable

Reasons for refusal:_________________________________________

--------------------------------------------

**OBSERVATIONS**

Informal conversations (*Use this space to note down all informal clarifying conversations that took place during the visit, summarising the content of each Conversation.*): _________________________________________

General observations (*Note down the NPO’s general impressions*): _________________________________________

SIGNATURES

Observer name: __________________ Code: ___ Signature: _______________________ Date: __-__-____
